# Genetically engineered lymphocytes in metastatic melanoma: TIL 1383I TCR transduced T-cells are detectable after infusion

**DOI:** 10.1186/2051-1426-1-S1-P31

**Published:** 2013-11-07

**Authors:** Courtney Regan, Michael Nishimura, Ann Lau Clark, Kelly Moxley, Gina Scurti, Vladimir Slepushkin, Andre Roy, Kathy Schonely, Boro Dropulic, Joseph Clark

**Affiliations:** 1 Cardinal Bernadin Cancer Center, Loyola University Medical Center, Maywood, IL, USA; 2 Lentigen Corporation, Gaithersburg, MD, USA

## Background

Previous studies in adoptive T-cell transfer have suggested that persistence of the transduced T-cells is central to making this therapy a viable option. Understanding the behavior of tumor-reactive T-cells in cancer patients and measuring persistence are two objectives in a phase I clinical trial using TCR TIL 1383I transduced T cells in stage IV melanoma patients.

## Methods

Peripheral blood mononuclear cells (PBMCs) were isolated from a melanoma patient, activated with anti-hCD3 with rhIL2 and rhIL15, transduced with lentivirus encoding the TIL 1383I TCR, and expanded to treatment numbers. 2 x 10^8^ transduced cells were suspended in 5% human albumin and infused over 30 minutes. The infusion was preceded by lymphodepletion with fludarabine and cyclophosphomide and followed with low dose IL-2 for one week. A modified CD34 cassette in the vector enabled monitoring of the transduced T cells in the patient’s PBMC post-infusion. PBMC were collected from patient on days 1, 3, 5, 7, 14, 25, and 35. The presence of transduced T cells at each timepoint was measured by staining with anti-CD34 mAb and analyzed using a BD LSRFortessa flow cytometer.

## Results

Transduced T cells were detected in the patient’s blood at day 25 post infusion. 1.45% of the patients T cells were TIL 1383I TCR transduced T-cells. We estimate that at least 10% of the infused TIL 1383I TCR transduced T cells were present after 4 weeks.

## Conclusion

Previous studies with TIL suggest better T-cell engraftment, persistence, and therapeutic efficacy with homeostatic proliferation after lymphodepletion. Out results confirm that the infused TIL 1383I TCR transduced T-cells could be detected 4 weeks after infusion. Localization of genetically engineered T-cells and ensuring their activation and function is of value in anti-tumor T-cell therapy.

